# Development, Optimization, and Validation of Forensic Analytical Method for Quantification of Anticholinesterase Pesticides in Biological Matrices from Suspected Cases of Animal Poisoning

**DOI:** 10.3390/toxics10050269

**Published:** 2022-05-23

**Authors:** André Rinaldi Fukushima, Juliana Weckx Peña-Muñoz, Luís Antônio Baffile Leoni, Maria Aparecida Nicoletti, Glaucio Monteiro Ferreira, Jan Carlo Morais Oliveira Bertassoni Delorenzi, Esther Lopes Ricci, Marlos Eduardo Brandão, Lorena de Paula Pantaleon, Vagner Gonçalves-Junior, Paula Andrea Faria Waziry, Paulo Cesar Maiorka, Helenice de Souza Spinosa

**Affiliations:** 1 Faculdade de Medicina Veterinária e Zootecnia, Universidade de São Paulo, São Paulo 05508-270, Brazil; pantaleon@usp.br (L.d.P.P.); vagnergjr@hotmail.com (V.G.-J.); maiorka@usp.br (P.C.M.); hspinosa@usp.br (H.d.S.S.); 2 Faculdade de Ciências da Saúde IGESP (FASIG), São Paulo 01301-000, Brazil; estherlopesricci@gmail.com (E.L.R.); brandao.marllos@gmail.com (M.E.B.); 3 CETAC—Centro de Treinamento Veterinário, São Paulo 01532-000, Brazil; weckxjuliana@gmail.com (J.W.P.-M.); leonifarmacia@gmail.com (L.A.B.L.); 4 Faculdade de Ciências Farmacêuticas, Universidade de São Paulo, São Paulo 05508-000, Brazil; nicoletti@usp.br (M.A.N.); gmf@usp.br (G.M.F.); 5 Centro de Ciências Biológicas e da Saúde, Universidade Presbiteriana Mackenzie, São Paulo 01302-907, Brazil; jan.bertassoni@mackenzie.br; 6 Western Atlantic University School of Medicine (WAUSM), Plantation, FL 33324, USA; paulaafariawaziry@gmail.com

**Keywords:** validation, intoxication, poisoning, n-methyl carbamates, organophosphates, metabolites, HPLC–DAD

## Abstract

Anticholinesterase pesticides are a main cause of the intentional or accidental poisoning of animals. Anticholinesterases include several substances that cause the overstimulation of both central and peripheral acetylcholine-dependent neurotransmission. Forensic analyses of poisoning cases require high levels of expertise, are costly, and often do not provide reliable quantitative information for unambiguous conclusions. The purpose of the present study was to develop and validate a method of high-performance liquid chromatography with diode array detector (HPLC–DAD) for the identification and quantitation of n-methyl carbamates, organophosphates and respective metabolites from biological samples of animals that were suspected of poisoning. HPLC–DAD is reliable, fast, simplistic and cost-effective. The method was validated for biological samples obtained from stomach contents, liver, vitreous humor and blood from four different animal species. The validation of the method was achieved using the following analytical parameters: linearity, precision, accuracy, selectivity, recovery, and matrix effect. The method showed linearity at the range of 25–500 μg/mL, and the correlation coefficient (r2) values were >0.99 for all matrices. Precision and accuracy were determined by the (a) coefficient of variation (CV), (b) relative standard deviation low-quality control (LQC), (c) medium-quality control (QCM), and (d) high-quality control (QCA). The indicated parameters were all less than 15%. The recovery of analytes ranged from 31 to 71%. The analysis of results showed no significant interfering peaks due to common xenobiotics or matrix effects. The abovementioned method was used to positively identify pesticide analytes in 44 of the 51 animal samples that were suspected of poisoning, demonstrating its usefulness as a forensic tool.

## 1. Introduction

Lethal intoxications are commonly related to the medicolegal area. A great challenge faced in both human and animal legal medicine is the determination of the cause and time of death. Often, such deaths result from intentional exposure to toxic agents. In 2012, the National System of Toxic-Pharmacological Information (SINITOX) reported 102,854 poisonings cases in Brazil involving humans and animals. Among the reported cases, 1199 (1.17%) poisonings involved animals.

Cases of the accidental exposure and intentional poisoning of animals via the administration of anticholinesterase pesticides have been reported in several countries [1,2,3,4]. Common pesticides include carbamates (CBs, e.g., aldicarb and carbofuran) and organophosphates (OPs, e.g., parathion), which are intended for domestic and agricultural needs. Exposure to these agents is of concern for veterinary medicine due to high levels of toxicity and lethality [5], accounting for approximately 30% of deaths among poisoned animals [6].

The mechanism of action of anticholinesterase pesticides involves the inactivation of the acetylcholinesterase (AChE) enzyme, which is responsible for the degradation of the neurotransmitter acetylcholine at cholinergic (either nicotinic or muscarinic) synapses. ACh works in both central and peripheral nervous systems, including neuromuscular junctions. When ACh is not degraded, it continues to depolarize post-synaptic membranes, resulting in the prolonged overstimulation of the nervous system and skeletal muscles [7]. Clinical signs of pesticide poisoning include diarrhea, vomiting, excessive salivation, miosis, airway constriction, bradycardia, frequent urination, muscle tremors, seizures, respiratory failure and death [7]. The development of the described signs depends on the pesticide dose and the animal’s size, age, species and overall health.

Several countries have implemented and often mandated regulations for the protection and welfare of animals. In Italy, for example, it is mandatory to report suspected animal poisoning cases to law enforcement agencies [8], and in the United States, the American Society for the prevention of Cruelty to Animals (ASPCA) has been involved in handling cases of potential toxicity since 1977 [9]. Compliance with such mandates has contributed to the rapidly emerging field of veterinary forensics, which in turn associates acts of cruelty to animals with violence against humans [10].

The use of aldicarb has been banned since October 2012 by the National Health Surveillance Agency (ANVISA), but this action has not yet reduced cases of aldicarb intoxication [11]. Aldicarb and carbofuran are classified as carbamates, which are reversible inhibitors of the enzyme acetylcholinesterase [12,13]. These substances are illegally used as rodenticides [14,15,16,17] and biological weapons [17,18,19,20,21]. Carbamates can be easily obtained by perpetrators who proceed to poison the target species, usually by mixing the toxins with canned food, meat, fish or inside sausages that are then consumed as a lethal dose [18,20,22,23]. By the time the animal is found, it is either dead or suffering from severe cholinergic clinical signs [13,24,25]. Vomiting and/or diarrheal contents are often found close to the animal, in addition to the remains of poisoned foods [18,20,22].

Forensic toxicological analyses are necessary for the identification of substances such as aldicarb, carbofuran, forate, and their metabolites due to their similar macroscopic appearance and necropsy findings. A list of commercially available anticholinesterase pesticides is shown in Table 1 [26].

Forensic science involves the study and application of laboratory methods to implications of criminal conducts and guidance for decision making that involves legal consequences. Forensics heavily relies on laboratory analyses of biological samples. Increased demand for forensic investigations and the need to ensure the high quality, reliability and reproducibility of results obtained through standardized equipment, as well as the comparability and traceability of analytes, are crucial for advancement of the field [27,28]. Obtaining false, unreliable, or biased data can lead to misleading conclusions, thus culminating in irreparable financial, academic, or judicial damages.

The process of method validation includes analytical merit figures such as linearity, sensitivity, limit of detection, limit of quantification, and precision [29,30]. Once the identification/quantification of analytes is achieved via a new technique, the method must be validated in order to guarantee performance, reproducibility and suitability for its intended use [31]. A strict validation process ensures that the method employed is objective, unambiguous and deemed reliable for providing useful information that can be used in decision making by regulatory agencies and the judiciary system [32].

The Society of Forensic Toxicologists [33] has established the necessary merit figures to perform the validation of quantitative analytical methods. Such figures are specificity/selectivity, limit of detection (LD), precision (intra-labor repeatability and/or inter-laboratory reproducibility), linearity, application interval, accuracy, recovery, uncertainty of measurement, and stability. Some parameters, such as the limit of quantification and robustness, can also be used for qualitative as well as quantitative analyses. These recommendations are in accordance and implemented by global regulatory agencies [33,34,35].

Separation chromatography techniques, such as gas chromatography (GC), high-performance liquid chromatography (HPLC) and capillary electrophoresis, are included in the repertoire of forensic methods that are widely used for toxicology chemical analysis [32]. Methods currently available for the identification and analysis of anticholinesterase pesticides, such as carbamate aldicarb, are based on gas and liquid chromatography [36,37]. These techniques provide qualitative data (the identification of molecules) and quantitative data (concentration measurements) derived from several types of sample matrices including the environment, foods, pharmaceuticals, biological origins, and many others [35,38]. However, the available methods are cumbersome, time-consuming, expensive, require highly specialized personnel, and are not as precise as HPLC chromatographical detection by diode array (DAD).

High-performance liquid chromatography with diode array detector (HPLC–DAD) is a method that allows for the precise identification of different metabolites via spectrum libraries [39]. Although DAD is scarcely used for the detection of pesticides, it is easier to perform and more affordable compared to liquid chromatography techniques coupled to mass spectrometry (HPLC–MS) or other techniques [40,41].

General protocols for chromatographic methods require the extraction of analytes from matrices, especially when testing complex samples such as foods, environmental matrices or biological samples from animal sources [42,43]. The widely used Quick Easy Cheap Effective Rugged (QuEChERS) extraction technique is based on the separation of analytes from sample proteins via the precipitation of the latter with acetonitrile, followed by sediment centrifugation and the direct usage of the supernatant, which contains the analytes for chromatographic detection [44,45,46]. In this sense, we chose to create a method inspired by this technique that contained similar characteristics.

The main goal of the present work was the development and validation of a novel forensic DAD chromatographic method that includes the optimal extraction, identification, and quantitation of AChE inhibitor pesticides that originated from several biological matrices of suspected poisoning cases. The applicability, accuracy, ease of operation, reliability, reproducibility and low-cost characteristics of the described HPLC–DAD method prove its versatility and usefulness in forensic laboratory routine analysis.

## 2. Materials and Methods

### 2.1. Pesticide and Matrix Standards

The following common cholinesterase inhibitors were selected as standard pesticide chemicals and used for the definition of performance criteria. The standard chemicals used for definition of performance criteria were: Aldicarb (Sigma^®^ San Luis, MO, USA), aldicarb–sulfoxide (Sigma^®^ ), aldicarb–sulfone (Sigma^®^), carbofuran and 3-OH-carbofuran (Sigma^®^), forate (Sigma^®^ San Luis, MO, USA), and forate–sulfoxide (Sigma^®^) compounds.

Standard matrix extracts were obtained from four different species (cats, dogs, rats, and chickens) that were free of pesticides and used for the establishment of protocol. The matrices were: whole blood, stomach contents, liver, kidneys, lungs, brains, and vitreous humor.

The xenobiotic-free matrices and suspected intoxication samples were obtained from the Department of Pathology (VPT), Pathology Service of the School of Veterinary Medicine and Animal Science of the University of São Paulo FMVZ/USP. This study was approved by the EMU ethics committee (CEUA Protocol No. 3006/2013) and (CEUA Protocol No. 2301/2011 and No. 3071/2013).

### 2.2. Equipment

The DAD chromatography procedure utilized a Shimadzu LC 20 A Prominence^®^ (Kyoto, Japan) liquid chromatograph equipped with a photodiode array (PDA) Shimadzu^®^ (Kyoto, Japan) detector, Shimadzu^®^ column oven, Shimadzu^®^ automatic injector, Shimadzu Lab Solutions integration system^®^ (Kyoto, Japan), and a Shimadzu vacuum degasser^®^ (Kyoto, Japan).

### 2.3. Chromatographic Conditions

For the determination of aldicarb, aldicarb, aldicarb–sulfoxide, aldicarb–sulfone, carbofuran, 3-OH-carbofuran, forate, and forate–sulfoxide, the chromatographic conditions were as follows: a φ3 µm shimpack^®^ (Kyoto, Japan) and a 100 Å C18 [22] column (150 mm × 4.6 mm) with a mobile phase of deionized water:acetonitrile in a gradient from 0% acetonitrile to 80% acetonitrile in 40 min; subsequently, a cleaning gradient was created starting at 40.01 min, reaching 100% acetonitrile in 45 min. Next, at 45.01 min, the initial condition was taken up with 0% acetonitrile, which remained for up to 50 min at 1.0 mL/min. Additionally, the column temperature was 35 °C, the injection volume was 10 μL, and the UV detection was at 213 nm for aldicarb and metabolites.

Adjustments were made to the column type, furnace temperature, mobile phase composition, mobile phase type, and mobile phase flow in order to optimize the technique. Fine-tuning was aimed to reduce the retention time and maintain the best peak resolution between the analytes of interest in comparison to the internal standard.

The interference check test was performed by evaluating the following xenobiotics: ivermectin, abamectin, dexamethasone, fipronil, atropine, prilocaine and amoxicillin at a concentration of 100 μg/mL, which is standard for drug detection.

### 2.4. Reagents and Solutions

The HPLC-grade compounds aldicarb (99.9%, *w*/*w*), aldicarb–sulfoxide (99.8%, *w*/*w*), aldicarb–sulfone (99.9%, *w*/*w*), carbofuran (99.9%, *w*/*w*), 3-OH-carbofuran (99.9%, *w*/*w*), forate (99.9%, *w*/*w*) and forate–sulfoxide (99.9%, *w*/*w*) were obtained from Sigma^®^ (San Luis, MO, USA), and acetonitrile was obtained from Merck^®^. HPLC-grade methanol was obtained from Merck^®^. For the extraction, chromatographic-grade chloroform (Merck^®^, Darmstadt, Germany), sodium chloride (Vetec^®^ Duque de Caxias, Brasil), anhydrous magnesium sulfate (Vetec^®^ Duque de Caxias, Brasil), anhydrous sodium sulfate (Vetec^®^), chromatographic-grade acetonitrile (Merck^®^), sodium phosphate (Vetec^®^) and sodium hydroxide (Vetec^®^ Duque de Caxias, Brasil) were used. Water was used from reverse osmosis type I and from the filtration system (Millipore^®^ Darmstadt, Germany). Consumables, glassware, and instruments included qualitative filter paper (Thermo Scientific^®^ Waltham, MA, USA), 0.22 μm syringe filters (Millipore^®^), glass beakers, glass filter funnels, 50 mL plastic tubes with screw caps (Thermo Scientific^®^), mechanical brand agitator (Daigger Scientific^®^ Hamilton, NJ, USA) multi-model microplate genie) and a refrigerated centrifuge (Eppendorf). Stock solutions with concentrations of 500.0 µg/mL of HPLC-grade aldicarb–sulfoxide and aldicarb–sulfone were individually prepared in acetonitrile (Merck^®^) and stored in a freezer at −80 °C. Standard dilutions containing aldicarb and metabolites were prepared from stock solutions. The internal standard used was terbuthylazine.

### 2.5. Preparation of the Fortified Samples

For the optimization and validation of the method, the biological matrices were as follows: whole blood, stomach contents, liver, kidneys, lungs, brains, and vitreous humor from four different animal species (cats, dogs, rats, and chickens) that were free of pesticides. Matrices were fortified with standard solutions containing the pesticide and metabolites at concentrations of 500, 250, 125, 62.5, and 31.25 µg/mL. The fortified water samples were used as the internal standard with terbuthylazine at a concentration of 100 µg/mL.

These concentrations were determined based on the following literature [11,20,23,47].

### 2.6. Application of the LLE and the Method Developed Inspired by QuEChERS

The optimized and validated method was applied for the determination of aldicarb, carbofuran and metabolites from the following biological matrices: whole blood, stomach contents, liver, kidneys, lungs, brains, and vitreous humor from four different animal species (cats, dogs, rats, and chickens). For validation purposes, the abovementioned matrices were fortified with the standards of aldicarb, carbofuran, and metabolite before being analyzed as trial runs to show the efficiency of the method prior to analysis of real samples.

### 2.7. Method for Validation

The optimized technique was validated under the consideration of the following parameters: selectivity, linearity, detection, lower and upper quantification limits, precision (repeatability and intermediary precision) and accuracy (recovery test and comparison of methods), according to recommendations of the Forensic Toxicology Laboratory Guidelines (SOFT) [33,35,48]. The accuracy of the method was evaluated, in three repetitions, with recovery tests at three concentration levels and with comparisons using the method inspired by the QuEChERS extraction method cited above [45].

### 2.8. Evaluation of Matrix Effect

Three calibration curves were constructed to evaluate the effect of each of the matrices. The determination of concentrations for the standard curves were based on clinically relevant concentrations that were estimated from cross-organism molecular modeling for pesticide harmfulness [49], and respective limits fell within detectable parameters of the equipment. Analytical curves were prepared as follows.

1. Aldicarb, carbofuran and its metabolites were solubilized in acetonitrile at different concentrations (25–1.000 µg/mL).
2. Aldicarb and metabolites were solubilized in whole blood, stomach contents, liver, kidneys, lung, brain, and vitreous humor from four different animal species (cats, dogs, rats, and chickens). Extracts were free of suspected pesticides (blank) and prepared at different standard concentrations (25–500 µg/mL). All samples received 10 µL of the terbuthylazine (100 µg/mL) internal standard solution.
3. Normal and putrefied matrices free of aldicarb and metabolites were fortified with different concentrations (25–500 µg/mL) of aldicarb and metabolites in acetonitrile to obtain the same final concentration relative to analytical curves 1 and 2 described above. These samples were submitted to the same extraction protocol.

### 2.9. Optimization of the Liquid–Liquid Extraction with the Method Inspired by Quick Easy Cheap Effective Rugged and Safe (QuEChERS)

The QuEChERS technique is well-established and widely used in food matrices [46]. Due to the fact that our biological matrices were derived from animal tissues, we chose to develop and validate an extraction process inspired by this technique. After some adjustments, the extraction that presented the best performance was the hybrid technique. It consisted of liquid–liquid extraction followed by the application of the QuEChERS-inspired extraction method for the further purification of the analyte.

The extraction principle was the use of an organic solvent-immiscible extractor in the matrix analysis based on the molecular oil/water partition coefficient. According to their lipophilicity, the analytes migrate (or not) to the oily portion after extensive agitation. To carry out the separation, a mixture of ether:chloroform (1:1) was used.

The QuEChERS-inspired technique was based on protein precipitation and the salting out effect, resulting from the first extraction of analytes from biological matrices using acetonitrile, magnesium sulfate, and sodium chloride with subsequent centrifugation and the use of the supernatant for direct chromatographic analysis.

The optimized method for determining carbamates in biological matrices consisted of extracting 5.00 g of each biological sample with a mixture of 1.0 g of NaCl in 1.00 mL of phosphate-buffered saline (PBS) and 10.0 ml of an ether:chloroform solution. This solution was kept in a shaker for 50 min for optimal extraction. Subsequently, the mixture was centrifuged at approximately 4000 g at 4 °C for 20 min. After centrifugation, the organic phase was filtered and the extract was dried at room temperature. The dry extract was resuspended in 1.0 mL of acetonitrile, to which 40 mg of MgSO4 and 10 mg of NaCl were added; then, the mixture was vigorously shaken and the supernatant was filtered through a 0.22 μm HPLC filter and directly analyzed by HPLC–DAD.

**Figure 1:**
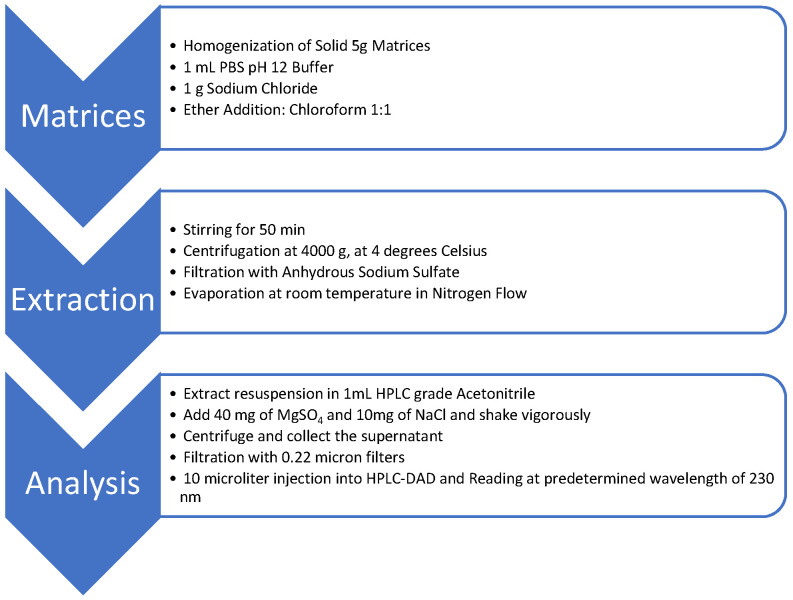
Flowchart for extraction and preparation of samples for analysis.

Optimized extraction is summarized in the flowchart in Figure 1.

### 2.10. Homogeneity (Fidelity)

In order to guarantee the homogeneity of the tests, the procedure of physical homogenization of the matrices was adopted, using a tissue homogenizer (mixer). All the analyzed tissues were thoroughly homogenized prior to being submitted to the extraction processes.

### 2.11. Rugged

Rugged tests were conducted via variations of the furnace temperature of the column by 1 °C and variations of the solvent marks, analyzer, and the column chromatographic mark used. None of these factors interfered and/or impaired the analysis. Other factors were not evaluated.

## 3. Results

### 3.1. Chromatographic Analysis

**Figure 2:**
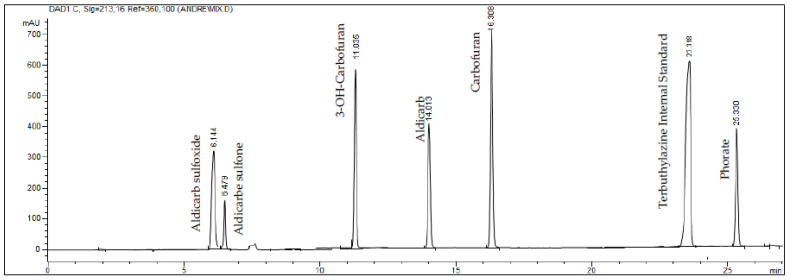
**Chromatogram of carbamates by HPLC–DAD**. Simultaneous analysis of carbamates shows distinct separation of peaks that correspond to each tested carbamate. Notice the high resolution for each principal component and the absence of background noise.

The optimized conditions for the simultaneous analysis of carbamates provided distinct separation peaks of the principal components, as shown in the chromatogram of Figure 2.

Changes were made to the column type, furnace temperature, mobile phase composition, mobile phase type, and mobile phase flow in order to optimize the technique. Fine-tuning was aimed to reduce the retention time and maintain the best peak resolution between the analytes of interest in comparison to the internal standard.

The peaks with retention times (tR) equal to 6.144, 6.479, 11.035, 14.013, 23.318, and 25.330 min corresponded to aldicarb–sulfoxide, aldicarb–sulfone, 3-OH-cabofuran, aldicarb, carbofuran, terbuthylazine (internal standard), and forate, respectively. An ultraviolet detector was selected for the simultaneous detection of the carbamates at the optimal absorption wavelength of 213 nm. The chromatographic interference check test showed no detection of any tested xenobiotics (ivermectin, abamectin, dexamethasone, fipronil, atropine, prilocaine and amoxicillin; data not shown).

The criterion for the choice of these analytes was their frequent and common use as drugs for animal therapy; therefore, there was a higher probability of finding these drugs in the sample’s matrices, which could have interfered with sample analysis.

### 3.2. Validation of the Optimized Method

#### 3.2.1. Detection and Quantification Limits

The HPLC–DAD detection limit [50] was determined as being equal to at least 3 times the baseline signal (noise) obtained for blank (water) samples. Calibration samples were fortified with carbamates (dilution range from 1 to 500 µg/mL) submitted to the LLE and QuEChERS-inspired extraction technique before analysis. The quantification limit (QL) was determined as being a signal at least 10 times greater than initial noise signal. The detection limits for aldicarb and for carbofuran and carbaryl were 10 and 5 µg/mL, respectively, and the quantification limits were 25 and 17 µg/mL, respectively. These concentration limits fully meet the demands of forensic analysis, since carbamate contamination falls within these limits when encountered in foods, drinks, biological and environmental matrices [47,51].

#### 3.2.2. Sensitivity and Linearity

The linearity of the PDA detector response was evaluated by injecting solutions of each of aldicarb and metabolite over a wide concentration range (from 25 to 500 µg/mL). Calibration curves were constructed for each analyte. The detector proved to be linear for the three studied compounds.

The operational range used was from 25 to 500 µg/mL. This was built using blank matrices as well as extraction products solubilized with pure solvent. The correlation coefficients were above 0.99 for all analytes.

To check the matrix effect, the equations that describe six analytical curves are presented in Table 2 (an analytical curve prepared by the dissolution of the aldicarb, carbofuran, and metabolites in acetonitrile; an analytical curve prepared by the dissolution of the carbamates in water extracts free of pesticides; and an analytical curve prepared from the superposed matrices).

These analytical curves and respective linear regression data indicated that the ultraviolet detector response was linear for the three analyzed compounds, having correlation coefficients greater than 0.99 for all three analytical curves. The sensitivity and linearity results are shown in Table 2, Table 3, Table 4, Table 5, Table 6 and Table 7.

Recently published manuscripts have demonstrated the effects of matrices on the determination of pesticide residues by gas chromatography [35,52,53]. These effects are primarily observed in more complex samples such as biological, food and drink matrices. The mechanism of the matrix effect on determination by high-performance liquid chromatography with ultraviolet and photodiode array detection is not widely used to date and the literature lacks reports related to the topic. Tables S1–S6, which are available in the Supplementary Materials, present the complete data of the calibration curves for aldicarb, aldicarb–sulfoxide, aldicarb–sulfone, carbofuran, and 3-OH-carbofuran for the analyzed matrices.

#### 3.2.3. Precision

The precision was evaluated by means of the low-quality control (LQC), medium-quality control (QCM) and high-quality control (HQC), in triplicate, considering the linear interval of the method (intraday and interrace). The coefficient of variation (CV) should not exceed 15%, except for the LLQ, for which a CV of less than 20% is accepted following the recommendations of the RDC n. 27 [34]. Table 6 shows the precision results, indicating that the coefficient of variation in all matrices studied in the LQC, MQC, and HQC complied with the used legislation that establishes up to 15% of variation for the LLQ and up to 20% for the other variables. The precision results are presented in Table 3, Table 4, Table 5, Table 6 and Table 7.

#### 3.2.4. Accuracy

The intra-assay and inter-assay accuracy was measured by evaluating three concentrations of the analytes (low, medium and high) within the linearity range of the method (in triplicate), being expressed by the relation between the experimentally determined mean concentration and the corresponding theoretical concentration, as suggested by RDC n. 27 [34]. Table 8 shows the results obtained from the different biological matrices. It can be noted that all parameters of the different matrices met the resolution that establishes up to 15% of variation for the LLQ and for the other variables up to 20%. The accuracy results are presented in Table 8, Table 9, Table 10, Table 11, Table 12 and Table 13.

### 3.3. Selectivity

**Figure 3:**
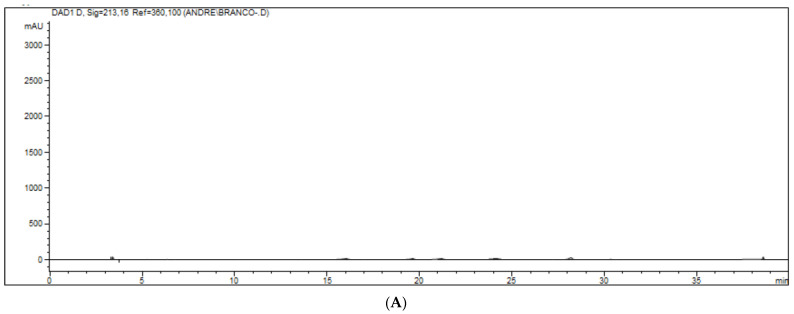

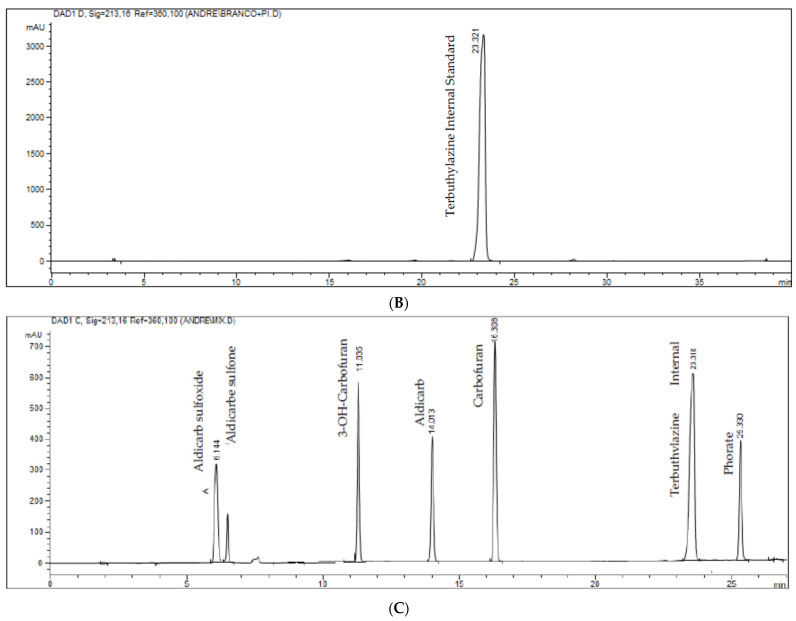
(**A**–**C**). Chromatograms of white samples represented in figure (**A**), zero (white sample added to the internal standard) represented in figure (**B**) and standards (aldicarb–sulfoxide, aldicarb–sulfone, aldicarb, 3-OH-cabrofuran, carbofuran, internal standard and phorate) represented in figure (**C**). The pattern was added to a pool of all matrices evaluated.

**Figure 4:**
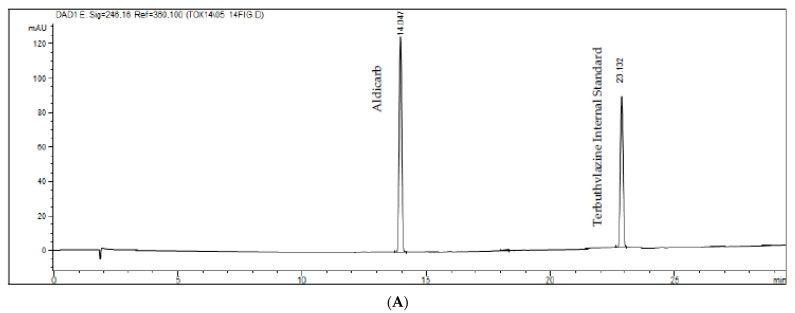

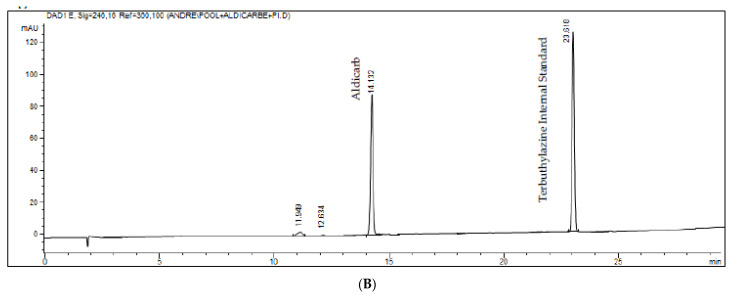
(**A**,**B**). Chromatograms of the white sample added from the internal standard and the aldicarb figure (**A**). Standard was added in a pool of all the matrices evaluated and the actual sample figure (**B**).

Selectivity was evaluated by analyzing matrices from four species obtained post-mortem. Each blank pool was confirmed using the same extraction procedures and the same chromatographic conditions as described above. The results were compared to those obtained with the standard alcoholic solution using a concentration close to the LIQ, as shown in Figure 3 and Figure 4. As acceptance parameters, a variation of 20% lower than the standard response was adopted in comparison to the standard response added to the matrices and 5% in the other evaluated concentrations, as established in ANVISA’s RDC 27 [34].

### 3.4. Recovery

This figure of merit evaluates the efficiency of the extraction procedure within a limit of variation. In the present study, the assay consisted of comparing the analytical results of samples extracted from three concentrations (low, medium and high) within the linearity range of the method with the results obtained using standard solutions, which reached 100% of the recovery. The recovery calculation was carried out as recommended by RDC n. 27 [34], in which the area ratio of the extracted and non-extracted standard is subtracted, with the result being expressed as a percentage. Table 14 shows the mean recovery (RM) in percentage obtained using the different biological matrices, indicating that the recovery range varied from 30 (heart) to 70% (stomach contents). These data suggest that stomach contents and blood are the most suitable matrices for the indication of presence of aldicarb; the other matrices can be used in the absence of the first ones. The recovery results are presented in Table 15.

### 3.5. Lower Limit of Quantification (LLQ) and Superior Limit of Quantification (SLQ)

The adopted LLQ was the lowest concentration of the calibration curve. The acceptance criteria for LLQ were expressed in CV form, as recommended by resolution RDC n. 27 [34]. Table 14, Table 15 and Table 16 show the CV (s) of the LLQ, as well as the CV means of the other points of the calibration curve (mean CV) and of the ISD in the different matrices. It can be noted that the CV of the LLQ in the different matrices was ≤15% according to the resolution. In addition, the mean CV was less than 20%, given the resolution, and the average CV of the IP presented a low variation (less than 5%).

The lower limit of quantification (LLQ) and the superior limit of quantification (SLQ) results are presented in Table 16, Table 17 and Table 18.

Following the development, optimization, and validation of the extraction method inspired by QuEChERS, it was applied to 172 matrices from samples submitted by the Pathology Services of the Department of Pathology of FMVZ/USP, as well as samples submitted by official organs. Carbamates are often illegally administered to intoxicate/poison animals. Carbamates are also known to be erroneously used as rodenticides and may lead to accidental or non-intentional intoxication. Thus, in the period from 2010 to 2015, it was possible to identify and quantify a total of 125 cases of carbamate pesticide and 47 cases of animal deaths that had no exposure to the toxic agents surveyed using the validated matrices. The methodology was shown to be equally efficient using the matrix effect.

### 3.6. Case Series of Suspected Intoxication Caused by Carbamate Pesticides in Animals Whose Tissue and Feed Samples Were Sent to LADTOX from 2010 to June 2015

Table 19 shows the case series of suspected intoxication caused by carbamate pesticides in animals whose tissue and feed samples were sent to LADTOX from 2010 to June 2015. It can be noted that the total samples analyzed in the period ranged from 12 to 48, and among the positive samples, the range was from 6 to 42.

From 2014 to June 2015, the described method was introduced to evaluate suspected cases of carbamate pesticide poisoning. Table 20 shows the results for this period, with materials 1–30 referring to the year 2014 and materials 31–51 referring to the year 2015. It can be noted that during this period, 86 materials were received in the LADTOX, 33 of were positive and 53 of which were negatives for aldicarb.

## 4. Discussion

The present work describes the development, validation and application of a novel HPLC–DAD method for the identification and quantitation of the n-methyl carbamate, organophosphate, and respective metabolite pesticides from biological samples of animals that were suspected of poisoning. The development of the method started with the theoretical study of the compounds under investigation. The analytes’ physicochemical and structural characteristics served as benchmarks for the selection of the chromatographic column, mobile phase, and main wavelengths for identification and quantification, which were aimed at acquiring optimal results for analysis.

The choice of matrices for method validation followed specific selection criteria that are routinely applied in forensics. (a) Blood is the most common and well-established fluid connective tissue that is used for forensic analysis. It is unanimously considered to be the matrix of choice for toxicological analysis since it contains information on biomarkers of organs functions and on introduced toxic agents and their modified byproducts. (b) Stomach contents are directly linked to the majority of intoxications that are orally administered via the ingestion of contaminated foods. Data obtained from stomach contents have important implications for the medicolegal involvement of veterinary medicine, which aids in determining whether intoxications were intentional or accidental. (c) The liver is a fundamental choice of solid tissue for post-mortem toxicology because it is the main site for the metabolism and biotransformation of drugs and toxins. The choice of this matrix represents an innovative approach for the analysis of aldicarb and its metabolites. Furthermore, the extent of biotransformation is indicative of a timeline from consumption to the time of death. (d) The choice of vitreous humor matrix presents another innovation in the field of veterinary toxicology because this matrix is not usually cited in forensic veterinary literature. The vitreous humor has advantages over other biological matrices because it allows for the detection of most xenobiotics and/or toxins, which are less prone to post-mortem redistribution. Simultaneously, substances detected in the vitreous humor show more stability over time after death [56]. (e) The other biological matrices used (lung, brain and kidney) served for the verification of post-mortem detection and distribution for testing whether these matrices could serve as references for analyses of poisoning by aldicarb.

The overall protocol was optimized with the intent of developing a simplistic and effective method of extraction and analysis that could meet all the respective validation guidelines and could be simultaneously applied to all the selected matrices. Planning a standardized extraction technique was particularly challenging because the matrices were very diversified. After several tests were performed, the extraction that yielded the best performance was a hybrid of a liquid–liquid extraction followed by the technique inspired by QuEChERS.

In the analysis of pesticides and multi-residues, the QuEChERS extraction method (which has this acronym because it is fast, easy, cheap, effective, robust and safe) has replaced less efficient traditional methods of extraction. The biggest advantage of this method is that the concentration steps can be dispensed with [57].

The QuEChERS technique is an established and widely used method for isolating pesticide residues from food matrices [46]. Since most of our biological samples can be classified as being derived from meat, the use of a method inspired by QuEChERS was be deemed appropriate for detection of pesticide residues from such matrices. In our extraction flow, as we are dealing with complex matrices, we chose to carry out an initial liquid–liquid extraction process using ether and chloroform, as described in Figure 1; after this extraction process, we used the method that was inspired by QuEChERS, with the modification of the proportional decrease in the amount of salts (40 mg of MgSO_4_ and 10 mg of NaCl), in addition to the fact that it was used as a purification technique for the first extraction.

The following figures of merit evaluated in the validation process were linearity performed by the internal standardization method: precision, accuracy, selectivity (residual and matrix effect), recovery, lower limit of quantification (LIQ), upper limit of quantification (LSQ), homogeneity (fidelity), stability (still in progress) and robustness. These tests were performed to verify the absence of co-occurrence with the evaluated standards. The criterion for the choice of xenobiotics was based on their frequent and common use as animal therapeutics, so there was a higher probability of finding the drugs in the sample matrices that could potentially interfere with the analysis of pesticides.

Curve linearity was obtained from triplicate analyses for aldicarb, aldicarb–sulfoxide, aldicarb–sulfone, carbofuran, 3-hydroxycarbofuran and forate for each of the biological matrices (whole blood, stomach contents, liver, kidneys, lung, brain, and vitreous humor). The linear regression equation (LRE) and the correlation coefficient (r2) for analytes are listed in the Supplementary Materials, Tables S1–S6.

Table 8, Table 9, Table 10, Table 11 and Table 12 detail method precision (%CV) and accuracy (% standard error (SE)) in processed biological matrices for both a single run (intra-assay) and three runs on different days (inter-assay) using standard curves of analytes.

Regarding the precision and accuracy for the method, it was possible to observe that the CV and SE did not exceed the maximum limit of 15% for any of the levels of carbofuran concentrations.

Our interference results indicated the appropriateness of the method for selectivity, which was verified by the low percentages of the response of interfering peaks in proximity to the analytes’ retention times (values were less than 20%). The interference responses peaks near the IS retention time were less than 5% [39,40].

Recovery liquid–liquid extraction can provide different recovery results, depending on the technique. Tennakoon et al. found recovery values from 77.3 to 91.7% for carbofuran in different biological matrices while employing extraction method inspired by QuEChERS in which the LOQ was 2.4 μg/mL [32]. Papoutsis et al. reported that the analyte recovery range obtained by using this extraction from blood matrix ranged from 88.9 to 103.1% for an LOQ of 0.015 μg/mL [41]. The recovery values obtained in the present work were comparable to the published data. Recovery data were determined from three different concentration levels and are shown in Table 13.

Regarding matrix effect, no significant interferences for the quantification of carbo- furan and 3-hydroxycarbofuran were observed

An NFIS equal to 1 proposed by Viswanathan et al. indicates that there is no significant interference from matrices [42]. It should also be noted that the matrix effect is dependent on the physicochemical properties of pesticides and characteristics of the endogenous materials [43,44].

For practical purposes, the application of the method presented here served the purpose of detecting trace pesticides from actual samples of suspected poisoning. The Toxicology Diagnostic Laboratory (LADTOX) of the School of Veterinary Medicine and Animal Science at the University of Sao Paulo is one of the reference laboratories for the Southeastern region of Brazil, mainly the State of Sao Paulo and its capital. Samples of stomach contents and liver from 62 animals, including dogs, cats, and other species suspected of poisoning, were referred to LADTOX from January 2015 to February 2017 and were subsequently tested for carbofuran and metabolite presence (Figure 3). Carbofuran was detected in 10 dogs (41.7%), 7 cats (30.4%), and 8 birds (80%), as listed in Table 7, which shows positive samples of carbofuran from stomach and/or liver.

The presented work corroborates a recent Italian study where the authors also used HPLC–DAD for the qualitative detection of carbofuran and other methyl-carbamates in baits and stomach contents for forensic veterinary toxicology. As with our quantitative method, the authors described the technique as effective, fast, and simple. In this context, 40% of the samples tested positive for aldicarb and carbofuran [45]. The Italian study and others have shown a higher proportion of positive poisoning cases among cats and dogs compared to other species [45,46]. These findings are in agreement with our results, which show a similar proportion of carbofuran poisoning found among dogs and cats. In addition, we also observed carbofuran poisoning among birds. This occurrence could be explained by bias due to low numbers of bird cases reported and/or investigated elsewhere. Furthermore, Brazil is home to one of the most diverse bird faunas in the world, which might increase the probability of poisoning and the number of suspected cases for testing [47].

The data found in the literature suggest a large variation in levels of carbofuran when different species and different matrices are compared. However, our results do not show such a discrepancy, indicating comparable levels of carbofuran found in the stomach contents and in liver samples of dogs and birds [29,30,32]. Further studies are needed for clarification of possible differences in sensitivity to pesticides, as well as other factors that could influence the discrepancy of forensic results.

## 5. Conclusions

Here, we developed and validated a method for the extraction and identification of anticholinesterase pesticides in biologicals matrices, whose merit figures met RDC n. 27 [34]. The method allowed for the extraction and identification of aldicarb and its metabolites (aldicarb–sulfoxide and aldicarb–sulfone), carbofuran and its metabolite (3-OH carbofuran), and forate and its metabolite (forato–sulfoxide). This method was applied to experimental samples that had been submitted to the pathology services. The method was proven efficient for the identification of aldicarb, aldicarb–sulfoxide, aldicarb–sulfone, carbofuran, 3-OH-carbofuran and forate. The present data indicate that all matrices employed in this study may be suitably used in veterinary forensic toxicology analysis to detect the presence of the indicated substances.

The applicability, ease of operation, and low-cost characteristics of the described method proves its usefulness for forensic laboratory routine analysis in the investigation of potential anticholinesterase pesticide accidental ingestion and/or poisoning cases.

## Figures and Tables

**Table 1:** Commercially available carbamates and organophosphates, adapted from [26].

Carbamates	Organophosphates	
Aldicarb	Acephate	Menazon
Aminocarb	Acetion	Merfos
Carbaryl	Cyanophos	Methamidophos
Carbofuran	Chlorthion	Ronel
Landrin	Crufomate	Temephos
Metacalmate	Phenithrotion	Tetrachlorvinphos
Methiocarb	Formotion	Propyl Thiopyrophosphate
Mexacarbate	Fostex	Tribufun
Propoxur	Iodofenphos	Trichlorfon
	Malathion	

Reproduced with permission from Caldas, L.Q.d.A., Intoxicações Exógenas Agudas por Carbamatos, Organofosforados, Compostos Bipiridílicos e Piretróides.; published by Centro de controle de intoxicações de Niteroi, 2000 [26].

**Table 2:** Precision in each matrix for aldicarb was evaluated by means of the coefficient of variation (CV%) of lower limit of quantification (LLQ), low-quality control (LQC), medium-quality control (MQC), and high-quality control (HQC).

Biological Matrices	LLQ%	LQC%	MQC%	HQC%
Blood	8.1	5.0	3.7	3.2
Stomach contents	5.9	5.5	8.5	0.6
Brain	4.3	5.1	5.4	3.4
Heart	15.0	20.0	15.3	13.9
Liver	10.2	3.9	8.6	1.5
Vitreous humor	9.3	5.6	4.5	4.6
Lung	4.0	4.5	8.6	4.2
Kidneys	6.0	4.7	7.5	5.6

**Table 3:** Variant coefficient of the calibration curves for aldicarb, aldicarb–sulfoxide, aldicarb–sulfone, carbofuran, and 3-OH-carbofuran for the analyzed matrices.

Matrices	Coefficient of Variation				
	Aldicarb	Aldicarb–Sulfoxide	Aldicarb–Sulfone	Carbofuran	3-OH-Carbofuran
Standard	0.12%	0.12%	0.15%	0.15%	0.20%
Blood	4.66%	3.65%	4.67%	2.53%	2.65%
Stomach contents	5.18%	2.16%	4.15%	2.07%	2.53%
Brain	4.70%	5.73%	3.72%	3.18%	4.10%
Heart	19.68%	18.38%	16.98%	2.81%	2.03%
Liver	7.28%	4.26%	6.48%	1.50%	3.35%
Vitreous humor	5.08%	2.18%	1.08%	1.22%	1.92%
Lung	4.57%	4.57%	3.27%	4.57%	8.68%
Kidneys	5.99%	4.99%	2.43%	5.99%	10.42%

**Table 4:** Precision in each matrix for aldicarb–sulfoxide was evaluated by means of the coefficient of variation (CV%) of the lower limit of quantification (LLQ), low-quality control (LQC), medium-quality control (MQC), and high-quality control (HQC).

Biological Matrices	LLQ%	LQC%	MQC%	HQC%
Blood	8.2	5.0	3.2	3.1
Stomach contents	6.0	5.1	8.1	0.4
Brain	4.1	5.4	5.2	3.1
Heart	14.3	19.1	15.1	13.0
Liver	10.1	4.1	8.4	1.1
Vitreous humor	9.4	5.2	4.2	4.2
Lung	4.1	4.3	8.3	4.1
Kidneys	6.2	4.5	7.3	5.1

**Table 5:** Precision in each matrix for aldicarb–sulfone was evaluated by means of the coefficient of variation (CV%) of the lower limit of quantification (LLQ), low-quality control (LQC), medium-quality control (MQC), and high-quality control (HQC).

Biological Matrices	LLQ%	LQC%	MQC%	HQC%
Blood	7.2	5.1	3.1	3.2
Stomach contents	4.0	5.2	8.0	0.5
Brain	4.4	5.1	5.0	3.2
Heart	12.3	16.1	14.1	10.0
Liver	9.1	4.2	8.5	1.1
Vitreous humor	8.2	5.1	4.1	4.1
Lung	4.5	4.5	8.1	4.4
Kidneys	4.2	4.1	7.6	5.2

**Table 6:** Precision in each matrix for carbofuran was evaluated by means of the coefficient of variation (CV%) of the lower limit of quantification (LLQ), low-quality control (LQC), medium-quality control (MQC), and high-quality control (HQC).

Biological Matrices	LLQ%	LQC%	MQC%	HQC%
Blood	7.2	4.1	1.1	1.5
Stomach contents	4.3	1.2	5.0	0.7
Brain	4.1	5.1	2.0	1.4
Heart	11.3	12.1	11.1	11.0
Liver	6.1	3.2	8.1	2.6
Vitreous humor	8.4	4.1	4.2	6.2
Lung	1.5	1.5	4.1	2.1
Kidneys	2.2	6.1	1.6	4.2

**Table 7:** Precision in each matrix for 3-OH- carbofuran was evaluated by means of the coefficient of variation (CV%) of the lower limit of quantification (LLQ), low-quality control (LQC), medium-quality control (MQC), and high-quality control (HQC).

Biological Matrices	LLQ%	LQC%	MQC%	HQC%
Blood	2.2	2.1	1.2	2.5
Stomach contents	6.3	3.2	3.1	0.4
Brain	2.1	3.1	2.4	1.2
Heart	10.1	11.5	4.1	9.0
Liver	3.5	4.2	3.1	3.6
Vitreous humor	3.4	2.1	2.2	3.2
Lung	3.5	2.5	2.1	2.4
Kidneys	2.5	3.5	1.5	2.2

**Table 8:** Lower limit of quantification (LLQ), low-quality control (LQC), medium-quality control (MQC), and high-quality control (HQC) calculated in each matrix, in triplicate, intracutaneous and intercurrent to aldicarb.

Biological Matrices	LLQ%	LQC%	MQC%	HQC%
Blood	6.97	2.16	11.34	3.2
Stomach contents	1.82	5.77	10.58	8.72
Brain	11.53	4.28	0.67	1.27
Heart	14.09	11.59	13.94	11.27
Liver	12.13	17.36	9.89	3.31
Vitreous humor	11.69	9.6	3.74	1.55
Lung	7.32	6.47	6.28	9.14
Kidneys	10.2	19.32	10.34	2.44

**Table 9:** Lower limit of quantification (LLQ), low-quality control (LQC), medium-quality control (MQC), and high-quality control (HQC) calculated in each matrix, in triplicate, intracutaneous and intercurrent to aldicarb–sulfoxide.

Biological Matrices	LLQ%	LQC%	MQC%	HQC%
Blood	5.63	1.14	12.32	2.22
Stomach contents	2.01	4.72	9.52	6.23
Brain	12.01	3.13	0.99	1.67
Heart	12.09	10.01	10.12	10.22
Liver	12.90	14.22	8.22	1.2
Vitreous humor	10.45	8.12	4.23	1.65
Lung	5.31	4.23	5.21	8.23
Kidneys	9.2	14.12	9.32	1.22

**Table 10:** Lower limit of quantification (LLQ), low-quality control (LQC), medium-quality control (MQC), and high-quality control (HQC) calculated in each matrix, in triplicate, intracutaneous and intercurrent to aldicarb–sulfone.

Biological Matrices	LLQ%	LQC%	MQC%	HQC%
Blood	4.22	2.41	10.25	1.55
Stomach contents	1.29	3.91	8.12	4.11
Brain	11.11	2.23	1.00	1.11
Heart	11.19	9.34	9.09	9.1
Liver	13.91	13.31	7.55	2.21
Vitreous humor	11.66	7.33	3.12	1.43
Lung	3.23	2.12	4.24	7.21
Kidneys	10.12	10.22	10.12	1.55

**Table 11:** Lower limit of quantification (LLQ), low-quality control (LQC), medium-quality control, (MQC), and high-quality control (HQC) calculated in each matrix, in triplicate, intracutaneous and intercurrent to carbofuran.

Biological Matrices	LLQ%	LQC%	MQC%	HQC%
Blood	5.67	3.22	9.45	2.87
Stomach contents	3.02	2.51	7.54	3.13
Brain	10.02	1.22	2.00	2.53
Heart	10.91	9.04	9.01	9.15
Liver	12.91	12.71	8.59	3.26
Vitreous humor	10.65	4.32	2.54	2.22
Lung	2.43	1.23	2.12	5.24
Kidneys	9.11	9.23	9.27	1.05

**Table 12:** Lower limit of quantification (LLQ), low-quality control (LQC), medium-quality control (MQC), and high-quality control (HQC) calculated in each matrix, in triplicate, intracutaneous and intercurrent to 3-OH-carbofuran.

Biological Matrices	LLQ%	LQC%	MQC%	HQC%
Blood	4.89	3.01	8.12	3.44
Stomach contents	2.11	2.32	6.23	3.08
Brain	9.01	1.76	1.65	1.99
Heart	9.08	10.23	10.01	10.11
Liver	11.23	11.12	7.29	1.24
Vitreous humor	9.35	3.33	1.66	1.99
Lung	1.42	2.31	3.54	4.22
Kidneys	8.12	7.53	8.17	0.92

**Table 13:** Presentation of mean recovery data in % of all analytes and different biological matrices [54].

Biological Matrices	Average Recovery of Adicarb	Average Recovery of Adicarb–Sulfoxide	Average Recovery of Adicarb Sulfone	Average Recovery of Carbofuran	Average Recovery of 3-OH-Carbofuran
Blood	60%	62%	61%	60%	64%
Stomach contents	70%	73%	72%	69%	70%
Brain	46%	50%	43%	47%	44%
Heart	30%	29%	29%	31%	33%
Liver	57%	66%	60%	53%	60%
Vitreous humor	32%	43%	40%	31	44%
Lung	31%	30%	32%	33%	29%
Kidneys	48%	32%	33%	50%	31%

**Table 14:** Coefficient of variation (CV) of the lower limit of quantification (LLQ) and the superior limit of quantification (SLQ), as well as the average CV (average of the CV of the other points of the calibration curve), and of the internal standard (ISD) in the different matrices, expressed in percentage of aldicarb–sulfone.

Biological Matrices	CV LLQ	CV Medium	CV Medium ISD	CV SLQ	CV Medium	CV Medium ISD
Blood	8.1%	4.7%	5.7%	3.8%	6.7%	2.4%
Stomach contents	5.9%	5.2%	2.6%	4.6%	8.1%	2.3%
Brain	4.3%	4.7%	7.8%	3.7%	3.0%	2.2%
Heart	15%	10.2%	11.7%	12.7%	11.6%	9.6%
Liver	10.2%	7.1%	11.5%	8.8%	8.0%	1.7%
Vitreous humor	9.3%	5.0%	9.9%	6.2%	3.6%	2.8%
Lung	4.0%	4.5%	3.9%	3.3%	6.2%	5.8%
Kidneys	6.0%	5.9%	7.2%	7.2%	8.9%	3.4%

**Table 15:** Coefficient of variation (CV) of the lower limit of quantification (LLQ) and the superior limit of quantification (SLQ), as well as the average CV (average of the CV of the other points of the calibration curve), and of the internal standard (ISD) in the different matrices, expressed in percentage of 3-OH-carbofuran.

Biological Matrices	CV LLQ	CV Medium	CV Medium ISD	CV SLQ	CV Medium	CV Medium ISD
Blood	4.1%	4.7%	2.0%	2.2%	6.7%	6.0%
Stomach contents	2.1%	5.2%	3.0%	5.7%	4.2%	5.0%
Brain	3.1%	4.7%	2.0%	4.2%	1.7%	3.0%
Heart	7.0%	10.2%	3.0%	12.2%	2.2%	2.0%
Liver	5.2%	7.1%	4.0%	5.3%	5.1%	1.0%
Vitreous humor	9.3%	5.0%	6.0%	2.5%	2.0%	4.0%
Lung	4.0%	4.5%	2.0%	5.1%	2.5%	2.0%
Kidneys	6.0%	5.9%	1.0%	3.4%	3.9%	2.0%

**Table 16:** Coefficient of variation (CV) of the lower limit of quantification (LLQ) and the superior limit of quantification (SLQ), as well as the average CV (average of the CV of the other points of the calibration curve), and of the internal standard (ISD) in the different matrices, expressed in percentage of aldicarb.

Biological Matrices	CV LLQ	CV Medium	CV Medium ISD	CV SLQ	CV Medium	CV Medium ISD
Blood	7.5%	3.6%	7.5%	3.2%	8.1%	4.7%
Stomach contents	3.9%	5.6%	9.5%	4.7%	5.9%	5.2%
Brain	7.9%	4.7%	3.0%	2.3%	4.3%	4.7%
Heart	14.5%	15.8%	14.6%	12.6%	15.0%	10.2%
Liver	11.2%	10.6%	9.2%	2.4%	10.2%	7.1%
Vitreous humor	10.5%	7.6%	4.1%	3.1%	9.3%	5.0%
Lung	5.7%	5.5%	7.4%	6.7%	4.0%	4.5%
Kidneys	8.1%	12.0%	8.9%	4.0%	6.0%	5.9%

**Table 17:** Coefficient of variation (CV) of the lower limit of quantification (LLQ) and the superior limit of quantification (SLQ), as well as the average CV (average of the CV of the other points of the calibration curve), and of the internal standard (ISD) in the different matrices, expressed in percentage of aldicarb–sulfoxide.

Biological Matrices	CV LLQ	CV Medium	CV Medium ISD	CV SLQ	CV Medium	CV Medium ISD
Blood	7.2%	3.3%	3.4%	3.1%	7.2%	2.2%
Stomach contents	6.0%	4.1%	4.8%	7.6%	4.9%	5.1%
Brain	2.1%	3.4%	3.1%	2.4%	2.1%	2.1%
Heart	15.0%	10.2%	4.0%	11.2%	15.0%	10.2%
Liver	10.2%	7.1%	4.0%	3.3%	10.2%	7.1%
Vitreous humor	9.3%	5.0%	5.0%	1.5%	9.3%	5.0%
Lung	4.0%	4.5%	4.0%	9.1%	4.0%	4.5%
Kidneys	6.0%	5.9%	5.0%	2.4%	6.0%	5.9%

**Table 18:** Coefficient of variation (CV) of the lower limit of quantification (LLQ) and the superior limit of quantification (SLQ), as well as the average CV (average of the CV of the other points of the calibration curve), and of the internal standard (ISD) in the different matrices, expressed in percentage of carbofuran.

Biological Matrices	CV LLQ	CV Medium	CV Medium ISD	CV SLQ	CV Medium	CV Medium ISD
Blood	4.0%	6.4%	3.7%	5.3%	2.2%	3.2%
Stomach contents	5.0%	3.7%	1.9%	6.3%	1.9%	8.7%
Brain	4.0%	7.1%	3.2%	2.0%	2.0%	1.2%
Heart	4.0%	11.1%	10.6%	10.1%	10.1%	11.2%
Liver	4.0%	9.5%	8.0%	8.3%	2.9%	3.3%
Vitreous humor	5.0%	9.5%	4.2%	3.4%	4.2%	1.5%
Lung	4.0%	2.0%	1.4%	3.1%	3.7%	9.1%
Kidneys	5.0%	5.7%	7.7%	5.4%	2.6%	2.4%

**Table 19:** Cases of suspected poisoning by carbamate pesticides (aldicarb and carbofuran) in animals whose tissue and feed samples were sent to the Laboratory of Toxicological Diagnosis (LADTOX) from 2010 to June 2015.

Samples			Years			
	2010	2011	2012	2013	2014	2015
**Dogs**	12	11	11	18	07	07
Positive	07	07	08	12	06	04
Negative	04	11	03	06	01	03
**Cats**	05	25	11	22	19	03
Positive	05	22	06	16	14	02
Negative		03	05	06	05	01
**Ration**		01	01		01	
Positive		01			01	
Negative			01			
**Birds**		08				
Positive		08				
Negative						
**Cattle**	01		01		01	01
Positive					01	
Negative	01		01			01
**Others**		01 ^a^	01 ^b^			
Positive		01	01			
Negative			-			
**Total**	18	48	26	40	28	12
Positive	12	42	14	28	23	06
Negative	06	06	12	12	05	06

^a^ skunk, ^b^ horse. Source: Reproduced with permission from Fukushima, A.R., Development of Analytical Methods Applied to Forensic Purpose Veterinary Forensics: Emphasis on Identifying Anticholinesterase Agents; published by Universidade de São Paulo, São Paulo, 2015 [55].

**Table 20:** Cases of suspected poisoning by the pesticide aldicarb in animals whose tissue and feed samples were sent to the Laboratory of Toxicological Diagnosis (LADTOX) from 2014 to June 2015.

Material	Stomach Contents	Liver	Blood	Ration	Species
1	-	+	-	+	Dog
2	-	-	-	-	Cat
3	-	+	-	-	Cat
4	-	-	-	-	Cat
5	-	-	-	-	Dog
6	+	+	-	-	Cat
7	-	-	+	-	Dog
8	+	-	-	-	Cat
9	-	-	-	-	Dog
10	+	-	-	-	Cat
11	-	-	-	-	Dog
12	+	+	-	-	Cat
13	+	+	-	-	Cat
15	+		-	-	Cat
16			-	+	Dog
17	-	-	-	-	Cat
18	+	+	-	-	Dog
19	-	-	-	-	Cat
20	+	+	-	-	Cat
21	-	-	-	-	Cat
22	+	+	-	-	Cat
23	+	-	-	-	Cattle
24	-	-	-	-	Cat
25	+	-	-	-	Cat
26	+	-	-	-	Cat
27	+	-	-	-	Cat
28	+	-	-	-	Cat
29	+	-	-	-	Dog
30	+	-	-	-	Skunk
31	-	-	-	-	Dog
32	-	-	-	-	Dog
33	-	-	-	-	Cat
34	+	+	-	-	Dog
35	+	-	-	-	Dog
36	-	-	-	-	Dog
37	-	-	-	-	Dog
38	-	-	-	-	Dog
39	-	-	-	-	Cattle
40	-	+	-	-	Cat
41	-	-	-	-	Dog
42	-	-	-	-	Dog
43	-	-	-	-	Cat
44	-	-	-	-	Cat
45	+	-	-	+	Dog
46	-	-	-	-	Cat
47	-	-	-	-	Cat
48	-	-	-	-	Chicken
49	-	-	-	-	Chicken
50	-	-	-	-	Chicken
51	-	-	-	-	Dog
Positive	19	10	01	03	
Negative	25	26	02	0	
**Total**	**44**	**36**	**03**	**03**	

+ positive for aldicarb; − negative for aldicarb. Source: Reproduced with permission from Fukushima, A.R., Development of Analytical Methods Applied to Forensic Purpose Veterinary Forensics: Emphasis on Identifying Anticholinesterase Agents; published by Universidade de São Paulo, São Paulo, 2015 [55].

## Data Availability

Not applicable.

## Supplementary Materials

The following supporting information can be downloaded at https://www.mdpi.com/article/10.3390/toxics10050269/s1, Table S1: Mean of the line equations, correlation coefficient (r2) and variant coefficient of the calibration curves for aldicarb for the analyzed matrices. Table S2: Mean of the line equations, correlation coefficient (r2) and variant coefficient of the calibration curves for aldicarb-sulfoxide for the analyzed matrices. Table S3: Mean of the line equations, correlation coefficient (r2) and variant coefficient of the calibration curves for aldicarb-sulfone for the matrices studied. Table S4: Mean of the line equations, correlation coefficient (r2) and variant coefficient of the calibration curves for carbofuran for the matrices studied. Table S5: Mean of the line equations, correlation coefficient (r2) and variant coefficient of the calibration curves for 3-OH-Carbofuran for the matrices studied. Table S6: Mean of the line equations, correlation coefficient (r2) and variant coefficient of the calibration curves for 3-OH-Carbofuran for the matrices studied.
