# Supplementary material for: Development, Optimization, and Validation of Forensic Analytical Method for Quantification of Anticholinesterase Pesticides in Biological Matrices from Suspected Cases of Animal Poisoning
Source: Toxics. 2022 May 23;10(5):269. doi: 10.3390/toxics10050269 (PMC9144076; doi:10.3390/toxics10050269)
Supplement: Supplementary file 1 [file toxics-10-00269-s001.zip › toxics-1675017-SI.pdf]

toxics

MDPI

Supplementary Materials: Development, Optimization, and Validation of Forensic Analytical Method for Quantification of Anticholinesterase Pesticides in Biological Matrices from Suspected Cases of Animal Poisoning

André R. Fukushima, Juliana Weckx Peña-Muñoz, Luís A. B. Leoni, Maria A. Nicoletti, Glaucio M. Ferreira, Jan C. O. B. Delorenzi, Esther L. Ricci, Marlos E. Brandão, Lorena P. Pantaleon, Vagner Gonçalves-Junior, Paula Andrea Faria Waziry, Paulo C. Maiorka and Helenice S. Spinosa

Table S1. Mean of the line equations, correlation coefficient (r^2) and variant coefficient of the calibration curves for aldicarb for the analyzed matrices.

Matrices	Mean Line Equation	r^2 Medium	Coefficient of Variation
Standard	y = 0.0011x – 0.0022	0.99	0.12%
Blood	y = 0.0011x – 0.0047	0.99	4.66%
Stomach contents	y = 0.0011x + 0.0107	0.99	5.18%
Brain	y = 0.0011x + 0.0056	0.99	4.70%
Heart	y = 0.001x + 0.0074	0.99	19.68%
Liver	y = 0.0014x – 0.0403	0.99	7.28%
Vitreous humor	y = 0.0012x + 0.0142	0.99	5.08%
Lung	y = 0.0011x + 0.0241	0.99	4.57%
Kidneys	y = 0.0012x + 0.013	0.99	5.99%

Table S2. Mean of the line equations, correlation coefficient (r^2) and variant coefficient of the calibration curves for aldicarb-sulphoxide for the analyzed matrices.

Matrices	Mean Line Equation	r^2 Medium	Coefficient of Variation
Standard	y = 0.0002x – 0.0012	0.99	0.12%
Blood	y = 0.0002x – 0.0015	0.99	3.65%
Stomach contents	y = 0.0002x + 0.0119	0.99	2.16%
Brain	$y = 1 \times 10^{-0.4}x + 0.0144$	0.99	5.73%
Heart	y = 0.0002x – 0.0097	0.99	18.38%
Liver	$y = 9 \times 10^{-0.6}x + 0.0034$	0.99	4.26%
Vitreous humor	$y = 3 \times 10^{-0.5}x + 0.0008$	0.99	2.18%
Lung	y = 0.0011x + 0.0241	0.99	4.57%
Kidneys	$y = 4 \times 10^{-0.5}x + 0.0023$	0.99	4.99%

Table S3. Mean of the line equations, correlation coefficient (r^2) and variant coefficient of the calibration curves for aldicarb-sulphone for the matrices studied.

Matrices	Mean line equation	r^2 medium	Coefficient of variation
Standard	y = 0.0016x + 0.0449	0.99	0.15%
Blood	y = 0.0017x + 0.0549	0.99	4.67%
Stomach contents	$y = 7 \times 10^{-0.6}x + 0.0002$	0.99	4.15%
Brain	$y = 1 \times 10^{-0.5}x + 0.0005$	0.99	3.72%
Heart	$y = 7 \times 10^{-0.5}x + 0.0085$	0.99	16.98%
Liver	$y = 6 \times 10^{-0.6}x + 4 \times 10^{-0.5}$	0.99	6.48%
Vitreous humor	$y = 5 \times 10^{-0.6}x – 0.0001$	0.99	1.08%
Lung	$y = 5 \times 10^{-0.6}x + 0.0002$	0.99	3.27%

Kidneys	y = 1 × $10^{-0.5}$x + 0.0006	0.99	2.43%

Table S4. Mean of the line equations, correlation coefficient (r^2) and variant coefficient of the calibration curves for carbofuran for the matrices studied.

Matrices	Mean Line Equation	r^2 Medium	Coefficient of Variation
Standard	y = 0.0005x – 0.0077	0.99	0.15%
Blood	y = 0.0004x – 0.0064	0.99	2.53%
Stomach contents	y = 0.0004x + 0.0037	0.99	2.07%
Brain	y = 0.0004x – 0.0025	0.99	3.18%
Heart	y = 0.0003x + 0.0081	0.99	2.81%
Liver	y = 0.0003x – 0.0069	0.99	1.50%
Vitreous humor	y = 0.0003x + 0.0002	0.99	1.22%
Lung	y = 0.0011x + 0.0241	0.99	4.57%
Kidneys	y = 0.0012x + 0.013	0.99	5.99%

Table S5. Mean of the line equations, correlation coefficient (r^2) and variant coefficient of the calibration curves for 3-OH-Carbofuran for the matrices studied.

Matrices	Mean Line Equation	r^2 Medium	Coefficient of Variation
Standard	y = 0.0003x – 0.0007	0.99	0.20%
Blood	y = 0.0003x – 0.0011	0.99	2.65%
Stomach contents	y = 0.0003x + 0.0013	0.99	2.53%
Brain	y = 0.0003x + 0.0023	0.99	4.10%
Heart	y = 0.0002x + 0.0064	0.99	2.03%
Liver	y = 0.0002x + 0.0053	0.99	3.35%
Vitreous humor	y = 0.0002x + 0.0074	0.99	1.92%
Lung	y = 0.0002x + 0.0093	0.99	8.68%
Kidneys	y = 0.0002x + 0.0078	0.99	10.42%

Table S6. Mean of the line equations, correlation coefficient (r^2) and variant coefficient of the calibration curves for 3-OH-Carbofuran for the matrices studied.

Matrices	Mean Line Equation	r^2 Medium	Coefficient of Variation
Standard	y = 0.0003x – 0.0007	0.99	0.20%
Blood	y = 0.0003x – 0.0011	0.99	2.65%
Stomach contents	y = 0.0003x + 0.0013	0.99	2.53%
Brain	y = 0.0003x + 0.0023	0.99	4.10%
Heart	y = 0.0002x + 0.0064	0.99	2.03%
Liver	y = 0.0002x + 0.0053	0.99	3.35%
Vitreous humor	y = 0.0002x + 0.0074	0.99	1.92%
Lung	y = 0.0002x + 0.0093	0.99	8.68%
Kidneys	y = 0.0002x + 0.0078	0.99	10.42%

Filename:	toxics-1675017-SM.docx
Directory:	E:\5.23\metals-1712071
Template:	C:\Users\MDPI\Desktop\Toxics\materials\toxics-template.dot
Title:	Type of the Paper (Article
Subject:	
Author:	MDPI
Keywords:	
Comments:	
Creation Date:	8/24/2021 2:59:00 PM
Change Number:	9
Last Saved On:	5/23/2022 5:34:00 PM
Last Saved By:	MDPI
Total Editing Time:	20 Minutes
Last Printed On:	5/23/2022 5:34:00 PM
As of Last Complete Printing	
Number of Pages:	2
Number of Words:	724
Number of Characters:	3,363
